# The differential expression of miRNAs between ovarian endometrioma and endometriosis-associated ovarian cancer

**DOI:** 10.1186/s13048-020-00652-5

**Published:** 2020-05-02

**Authors:** Natsuho Nakamura, Yoshito Terai, Misa Nunode, Kana Kokunai, Hiromi Konishi, Sayaka Taga, Mayumi Nakamura, Masae Yoo, Masami Hayashi, Yoshiki Yamashita, Masahide Ohmichi

**Affiliations:** 1 Department of obstetrics and gynecology, Osaka Medical College, 2-7 Daigaku-machi, Takatsuki, Osaka, 569-8686 Japan; 2 Department of Obstetrics and Gynecology, Kobe University Graduate School of Medicine, Kobe, Japan; 3 Hirakata City Hospital, Osaka, Japan; 4 Umeda Fertility Clinic, Osaka, Japan

**Keywords:** miRNA microarray, miR-486-5p, Ovarian endometrioma, Endometriosis-associated ovarian cancer

## Abstract

**Background:**

MicroRNAs (miRNAs) have been implicated to play a vital role in development, differentiation, cell proliferation and apoptosis. However, which miRNAs are actually associated with endometriosis-associated ovarian cancer remains controversial.

**Methods:**

Serum and ascites samples were obtained from all patients. Serum samples from 5 cases of ovarian endometrioma and endometriosis-associated ovarian cancer each were submitted for comprehensive miRNA microarray profiling. We investigated the differential expression of miRNAs between the two groups to confirm the pivotal role of miRNAs. Quantitative reverse transcription-polymerase chain reaction validation of five selected miRNAs [miR-92a-3p, miR-486-5p, miR-4484, miR-6821-5p, and miR-7108-5p] was performed, and miR-486-5p expression analysis was followed by proliferation and wound healing assays, depending on the expression of miR-486-5p.

**Result:**

miR-486-5p expression in serum and ascites samples from endometriosis-associated ovarian cancer patients was significantly higher than that from ovarian endometrioma patients. Moreover, the miR-486-5p level in serum and ascites samples was significantly correlated with the severity of the endometriosis. The upregulation of miR-486-5p in immortalized ovarian endometrioma cells significantly increased proliferation and migration. In contrast, the downregulation of miR-486-5p in these cells significantly decreased proliferation and migration.

**Conclusion:**

miR-486-5p might function as an oncogenic miRNA in endometriosis-associated ovarian cancer and could be a noninvasive biomarker to prospect the severity of ovarian endometrioma.

## Introduction

Sampson et al. reported the first case of suspected malignant change in endometriosis in 1925 and described that endometrial ovarian cancer may develop from endometriotic tissue based on microscopic findings [1]. Although the association between endometriosis and epithelial ovarian cancer has been epidemiologically and histopathologically investigated, the basic molecular and cellular involvements remain unknown.

Endometriosis-associated ovarian cancers (EAOC) include epithelial ovarian cancer (EOC), such as clear cell and endometrioid ovarian carcinoma [2, 3], and several studies have shown that the clinical characteristics and prognosis of EAOC are different from those of EOC [4].

There are two main risk factors for EOC, classified as either genetic or non-genetic factors. Genetic factors involve gene mutations, especially the hereditary factors from germline and somatic mutations in BRCA1 and BRCA 2 of high grade serous carcinoma EOC which are conveyed to the daughter by incomplete penetrance [5]. Smoking, obesity, contraception, high fat diet, infertility, endometriosis and co-morbid diseases are considered to be the non-genetic causes of EOC [6]. Most epidemiological studies have revealed that endometriosis increases the risk of ovarian cancer, but this association remains controversial. Recently, microRNAs have been shown to be engaged in the pathogenesis of endometriosis. MicroRNAs are a class of small, non-coding regulatory RNAs (18–25 nucleotides) that play a pivotal role in the regulation of post-transcriptional gene expression and are advocated to be included in diverse developmental and pathological processes [7]. MicroRNAs have been implicated to play a vital role in development, differentiation, cell proliferation and apoptosis. Circulating microRNAs in endometriosis have been shown to be potential biomarkers of the disease [8]**,** and Rekker K et al. reported that circulating miR-141, miR-200a and miR-200c have altered plasma levels in patients with endometriosis and vary with blood collection time [9]. Cosar et al. also revealed that miR-125b-5p can be a diagnostic marker for endometriosis and, in combination with miR-451a or miR3613-5b, improves the rate of a correct diagnosis of endometriosis [10]. However, which miRNAs are actually associated with EAOC remains controversial. Wu RL et al. showed miR-1, miR-133a and miR-451 were expressed lower in ovarian cancer compared with endometriosis; however, miR-141, miR-200a, miR-200c and miR-3613 were more highly expressed in ovarian cancer than in endometriosis [11]. Here we investigated the effect of miRNAs expression on the histopathological diagnosis in EAOC. Moreover, to confirm the pivotal role of miRNAs in the migration of ovarian cancer, we carried out wound healing and proliferation assays.

## Materials and methods

### Sample collection

Serum and ascites samples were obtained from a total of 41 patients who underwent surgical treatment for ovarian cancer or ovarian endometrioma at the Department of Obstetrics and Gynecology of Osaka Medical College between November 2016 and September 2018. This study of human samples was approved by the Institutional Review Board of the college (OMC-IRB 2060), and written informed consent was obtained from all patients participating in the study. The criteria for inclusion were no preoperative radiotherapy, chemotherapy, or hormone drug therapy, and no other inflammatory disease. The exclusion criteria included malignancy other than ovarian cancer, pregnancy or breastfeeding during the previous 6 months, infection disease, chronic or acute inflammatory disease, autoimmune disease, or cardiovascular disease. Endometriosis-associated ovarian cancer was confirmed to have ovarian cancer with associated foci of endometriosis in the same ovary by a gynecologic pathologist. Serum samples were collected at the induction of surgery, and ascites samples were collected immediately after the start of the surgery. Blood-contaminated ascites samples and lavage samples were excluded. No anticoagulant was used, and all samples were stored at − 80 °C until processing. Repeated defrosting was avoided during storage to ensure the quality of the samples.

### MicroRNA profiling

Serum samples from each of the 5 cases of ovarian endometrioma and endometriosis-associated ovarian cancer were submitted for comprehensive miRNA microarray profiling. These 10 cases in the miRNA array were just the first 5 cases of ovarian endometrioma and endometriosis-associated ovarian cancer, respectively. MicroRNA microarrays using the GeneChip miRNA 4.0 Array (Thermo Fisher Scientific, USA) were performed and analyzed by Filgen Biosciences and Nanosciences (Nagoya, Japan). Briefly, total RNA extracted from the serum samples was labeled using the FlashTag Biotin HSR RNA Labeling Kit (Thermo Fisher Scientific, USA) according to the manufacturer’s instructions. The array was then incubated using the GeneChip Hybridization Oven 645 (Thermo Fisher Scientific, USA) and washed using the GeneChip Fluidics Solution 450 (Thermo Fisher Scientific, USA) according to the manufacture’s protocol. The washed array was finally analyzed using the GeneChip Scanner 3000 7G (Thermo Fisher Scientific, USA).

### RNA extraction and quantitative real-time PCR

MicroRNA was extracted from serum and ascites samples with the mirVana PARIS Kit (Thermo Fisher Scientific, USA) according to the manufacturer’s recommendations. Cel-miR-39-3p mimic (Qiagen, Germany) was spiked in as exogenous control. Subsequent detection of miRNAs was carried out with TaqMan probes (miR-486-5p, cel-miR-39-3p) that were acquired from the TaqMan miRNA assay (Thermo Fisher Scientific, USA) and TaqMan PCR Master Mix (Thermo Fisher Scientific, USA) on the StepOne Plus Real Time PCR Detection System [Thermo Fisher Scientific, USA]. The expression level of miR-486-5p was assessed by the relative quantification method with cel-miR-39-5p as the reference gene (RQ = 2^-ΔΔCt^).

### Cell lines and cell culture

We used immortalized epithelial cells from ovarian endometrioma, EMOsis-CC/TERT [12], at the courtesy of Dr. Satoru Kyo (Shimane University, Shimane, Japan). The EMOsis-CC/TERT cells were immortalized by the combinatorial transfection of human *cyclinD1*, *cdk4* and human telomerase reverse transcriptase (*hTERT*) genes. The cells were grown in DMEM supplemented with 10% charcoal-stripped fetal bovine serum (FBS) (Equitech-Bio, Kerrville, TX) in an atmosphere of 5% CO2 at 37 °C.

### Upregulating or downregulating the expression of miR-486-5p in immortalized ovarian endometrioma cells

In order to upregulate the expression of miR-486-5p in the EMOsis-CC/TERT cells, they were transfected with Pre-miR miRNA Precursor/hsa-miR-486-5p or Pre-miR miRNA Precursor Negative Control by using the Lipofectamin RNAiMAX Transfection Reagent (Thermo Fisher Scientific, USA) and according to the manufacturer’s instructions. Conversely, in order to downregulate the expression of miR-486-5p in the EMOsis-CC/TERT cells, they were transfected with mirVana miRNA inhibitor/hsa-miR-486-5p or mirVana miRNA Inhibitor Negative Control by using the Lipofectamin RNAiMAX Transfection Reagent (Thermo Fisher Scientific, USA) and according to the manufacturer’s protocol.

### Cell miRNA expression analysis

TaqMan probes and primers [miR-486-5p, U6] were purchased from the TaqMan miRNA assay (Thermo Fisher Scientific, USA). Subsequent detection of miRNAs was carried out with the TaqMan PCR Master Mix on the StepOne Plus Real Time PCR Detection System (Thermo Fisher Scientific, USA). The expression level of miR-486-5p was assessed by using the relative quantification method, with U6 as the reference gene and was verified by comparison with the control cells

### Proliferation assay

For the detection of cell growth, the cells were seeded into a 96-well cell plate [2 × 10^4^ cells per well] and subjected to the MTS assay. Briefly, cells were transfected for 24 h, and the CellTiter 96 AQuenous (MTS) One Solution Reagent (Promega) was added to each well. The cells were then incubated for 1 h, and absorbance was recorded at 490 nm using the Corona SH-1000 lab absorbance microplate reader (Corona Electric, Ibaraki, Japan). The experiments were performed at least three times.

### Wound-healing assay

For the wound-healing assay, transfected cells were placed in a 6-well plate until the cells were confluent. One artificial wound per well was then scratched into the monolayer using a sterile, plastic, 200-μL micropipette tip to generate a uniform wound devoid of adherent cells. The cells were then washed three times with phosphate-buffered saline solution (PBS) and incubated in DMEM serum-free medium. Wound closure was monitored according to digital photographs taken with a phase-contrast microscope (Nikon Diaphot 300; Nikon, Tokyo, Japan) across the wound at the moment of wounding and at 12 and 24 h after wounding. The experiments were performed at least three times.

### Statistical analysis

Statistical analysis was made using the JMP software package (SAS Institute, Cary, NC), and statistical significance of difference was determined according to the Mann-Whitney U test or Spearman’s correlation coefficient, as appropriate. A *P* value of < 0.05 was considered to be statistically significant.

## Results

### Patients’ clinical and histopathological characteristics

The study included seven cases of endometriosis-associated ovarian cancer and 34 cases of ovarian endometrioma. Clinical and histopathological characteristics of EAOC and OE patients are listed in Table 1. The EAOC cases included four endometrioid carcinomas and three clear cell carcinomas. Age, tumor diameter, CA125 level and CA19–9 level were significantly higher in EAOC compared to those in OE.

**Table 1:** Table 1

	Endometriosis-Associated Ovarian Cancer [***n*** = 7]		Ovarian Endometrioma [***n*** = 34]	***P*** value
**Age [y]**	47.8 ± 9.78		38.0 ± 7.6	*p* < 0.01
**BMI**	21.8 ± 3.7		22.1 ± 3.9	n.p.
**Diameter [mm]**	155.7 ± 86.8		93.2 ± 41.6	*p* < 0.01
**Histologic type**	Endometrioid Carcinoma G1	2	Endometriotic cyst	
	G2	1		
	G3	1		
	Clear cell carcinoma	3		
**Clinical stage**	Stage I	5	–	
	II	1		
	III	1		
	IV	0		
**CA125 [U/ml]**	220.9 ± 269.4		87.3 ± 93.8	*p* < 0.01
**CA19–9 [U/ml]**	173.7 ± 296.1		39.6 ± 65.6	*p* < 0.01
**r-ASRM score**	–		59.4 ± 28.6	

Value are represented as mean ± SD [range]

Mann-Whitney U test

### Expression profiling of miRNAs

**Fig. 1:**
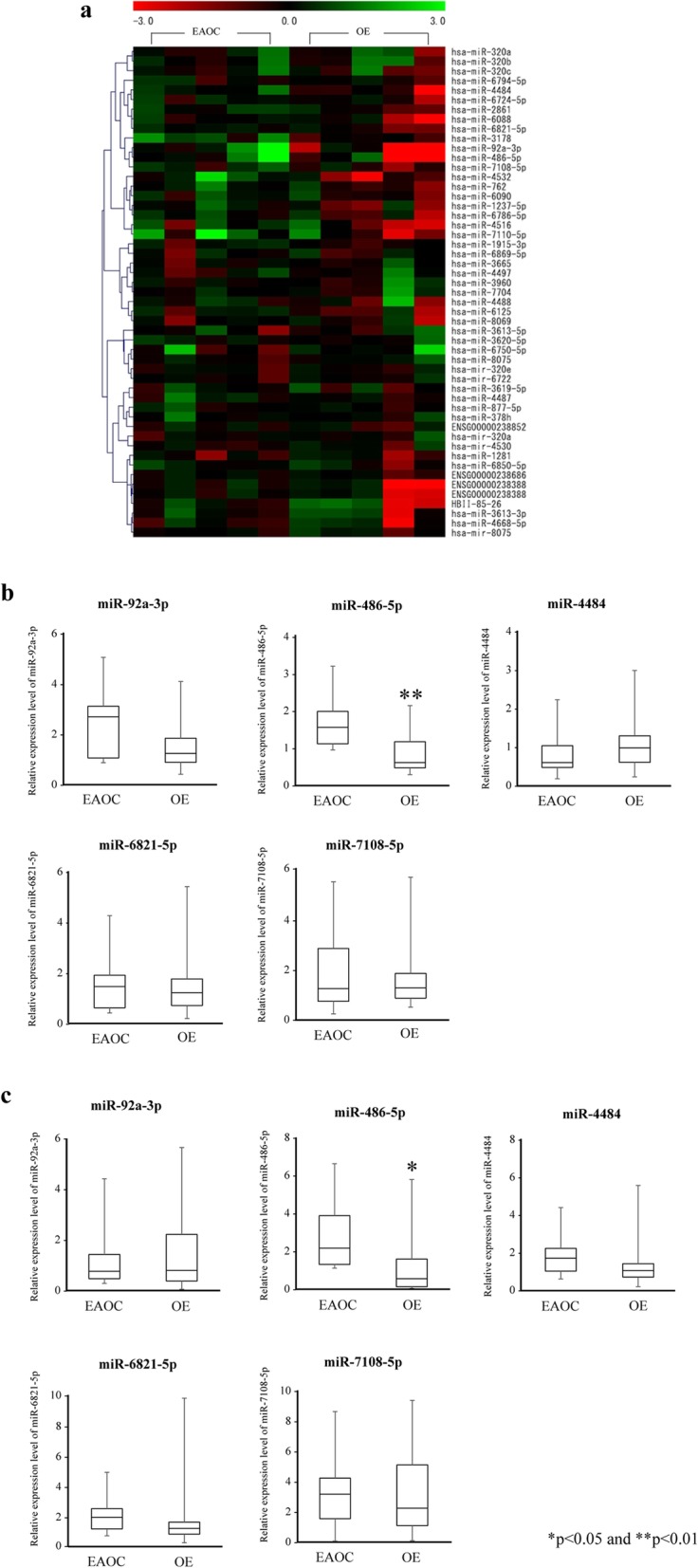
miRNA expression from EAOC and OE patients. **a:** Heat map summarizing the expression value of 51 miRNAs EAOC and OE patients. Color ranges from red [low expression] to green [high expression]. **b:** miRNA expression in serum samples from EAOC and OE patients. **c:** miRNA expression in ascites samples from EAOC and OE patients. * *p* < 0.05 and ** *p* < 0.01

Based on the median value of miRNA arrays from the 10cases, the expression ratio of each case was evaluated. We calculated whether the expression ration was greater than 2 or less than 0.5 for each group. The number of cases whose miRNA expression ratio was within this range was a total of 51 (Fig. 1a). Among these differentially expressed miRNAs, we chose five miRNAs that were pre-designed assays with a clear difference between the two groups. These included miR-92a-3p, miR-486-5p, miR-4484, miR-6821-5p, and miR-7108-5p, which we then further validated individually using quantitative reverse transcription-polymerase chain reaction (qRT-PCR) as presented below.

### MiR-486-5p was significantly increased in the serum and ascites of EAOC patients

**Fig. 2:**
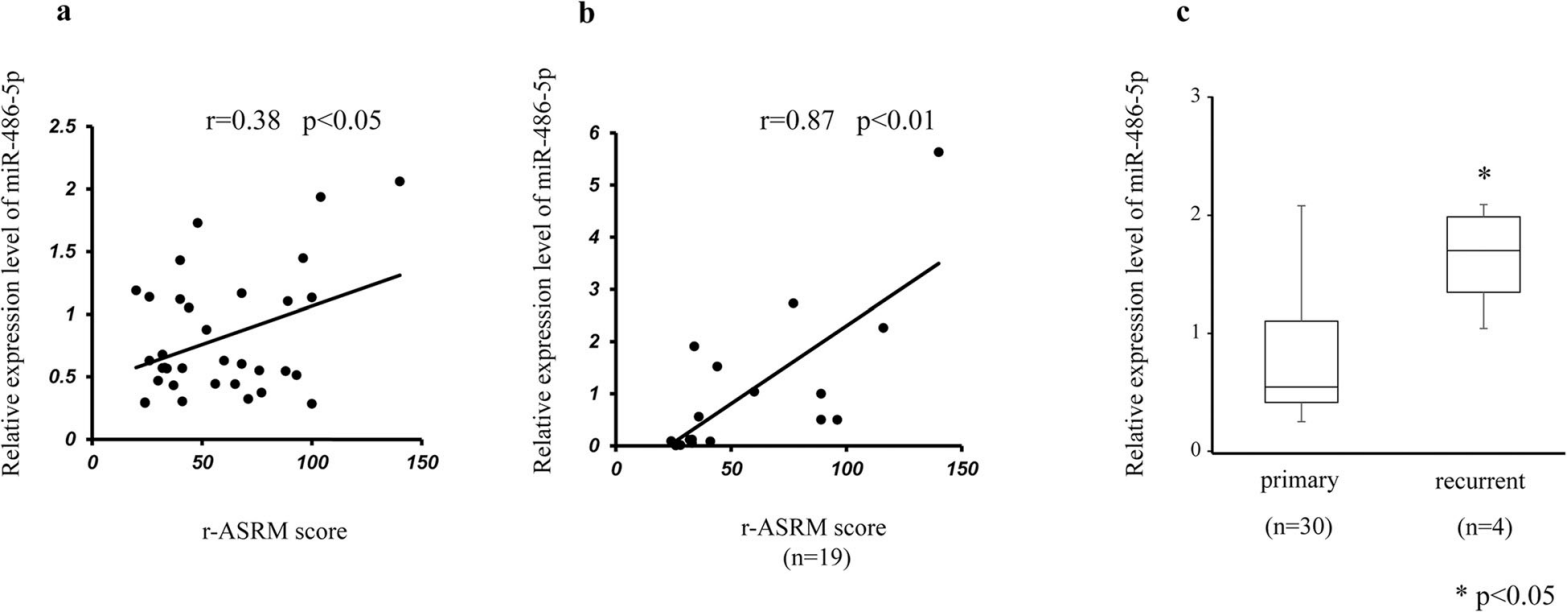
Comparison of miR-486-5p with clinical factors. **a**: The correlation of miR-486-5p expression in serum and r-ASRM score. **b**: The correlation of miR-486-5p expression in ascites and r-ASRM score. **c:** miR-486-5p expression in serum from primary and recurrent patients. * *p* < 0.05

We quantitatively analyzed expression levels of five candidate miRNAs in the serum and ascites samples from seven EAOC patients and 34 OE patients. Quantitative analysis of the five candidate miRNAs by qRT-PCR, consisting of miR-92a-3p, miR-486-5p, miR-4484, miR-6821-5p and miR-7108-5p, revealed significantly higher levels of miR-485-5p in serum and ascites samples from EAOC patients than in those from OE patients (Fig. 1b, c). No significant differences were observed in the serum and ascites levels of the remaining miRNAs between EAOC and OE patients. In order to determine whether higher serum and ascites miR-485-5p levels are associated with the severity of endometriosis, we analyzed correlations of their expression levels between the two samples. Spearman’s correlation coefficient indicated that the miR-486-5p level in serum and ascites samples was significantly correlated with the severity of the endometriosis (Fig. 2a, b). MiR-486-5p levels in serum were significantly higher in recurrent patients compared to primary OE patients (Fig. 2c). There was no correlation between the miR-486 expression ratio and patient’s age in the both EAOC and OE. The average age of EAOC patients was higher than that of OE; however, in all cases including OE and EAOC, no correlation was identified between the miR-486 expression ratio and patient’s age (Data not shown).

### Expression of miR-486-5p in immortalized ovarian endometrioma cells

**Fig. 3:**
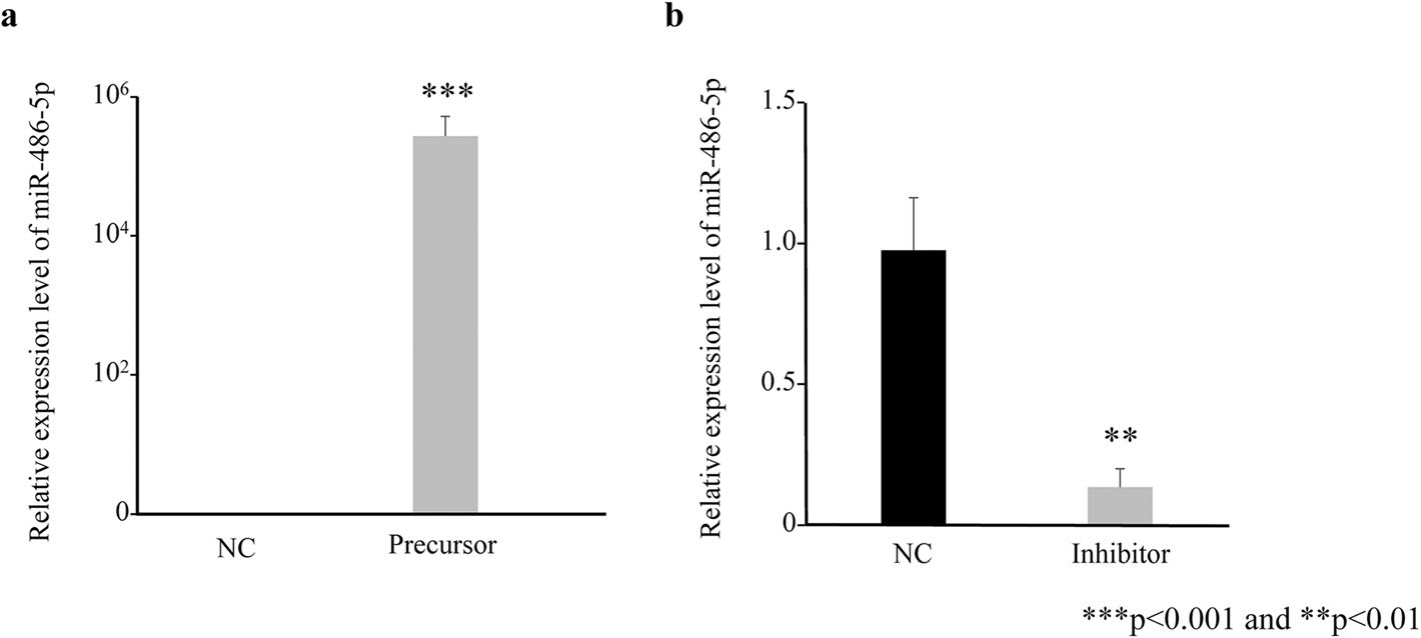
Expression of miR-486-5p in EMOsis-CC/TERT cells. **a:** miR-486-5p expression in EMOsis-CC/TERT cells transfected with miR-486-5p precursor or negative control. b: miR-486-5p expression in EMOsis-CC/TERT cells transfected with miR-486-5p inhibitor or negative control. ***p < 0.001 and **p < 0.01

The expression of miR-486-5p after transfection was measured using qRT-PCR to confirm that transfection could be performed reliably. Transfection of Pre-miR miRNA Precursor/hsa-miR-486-5p into EMOsis-CC/TERT cells (immortalized ovarian endometrioma cells) increased miR-486-5p expression, compared with cells transfected with Pre-miR miRNA Precursor Negative Control (Fig. 3a). In contrast, transfection of mirVana miRNA inhibitor/hsa-miR-486-5p into EMOsis-CC/TERT cells decreased miR-486-5p expression, compared with cells transfected with mirVana miRNA Inhibitor Negative Control (Fig. 3b).

### MiR-486-5p increased the proliferation and migration of immortalized ovarian endometrioma cells

**Fig. 4:**
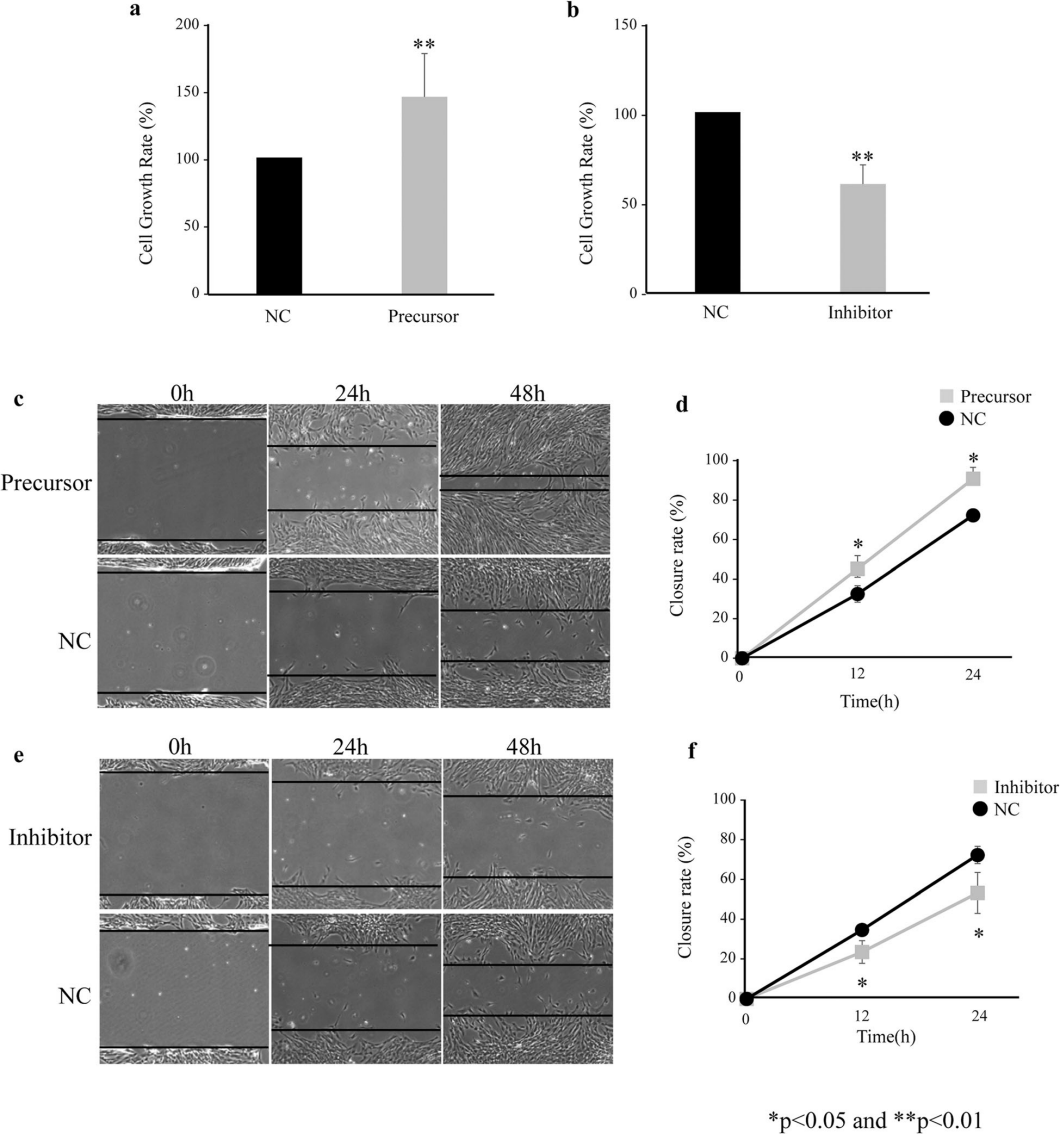
miR-486-5p promotes EMOsis-CC/TERT cells proliferation and migration in vitro. **a:** Cell proliferation assay comparing EMOsis-CC/TERT cells transfected with miR-486-5p precursor or negative control. **b**: Cell proliferation assay comparing EMOsis-CC/TERT cells transfected with miR-486-5p inhibitor or negative control. **c, d:** Wound healing assay comparing EMOsis-CC/TERT cells transfected with miR-486-5p precursor or negative control. **e, f:** Wound healing assay comparing EMOsis-CC/TERT cells transfected with miR-486-5p inhibitor or negative control. *p < 0.05 and **p < 0.01

Proliferation assaying showed that the upregulation of miR-486-5p by the transfection of miR-486-5p precursor significantly increased the proliferation rate of the EMOsis-CC/TERT cells, compared with the negative control group (Fig. 4a). In contrast, the downregulation of miR-486-5p by the transfection of miR-486-5p inhibitor significantly reduced the proliferation rate of the EMOsis-CC/TERT cells, compared with the negative control group (Fig. 4b).

We then examined the effect of miR-486-5p on migration and found that the upregulation of miR-486-5p in EMOsis-CC/TERT cells significantly increased migration (Fig. 4c, d). In contrast, the downregulation of miR-486-5p in EMOsis-CC/TERT cells significantly decreased migration (Fig. 4e, f).

## Discussion

To the best of our knowledge, this study is the first to explore the relationship between EOAC and miRNA 486-5p levels. It indicated that the miR-486-5p expression in blood and ascites in EAOC was higher than that in ovarian endometrial cyst and was positively correlated with the severity of the endometriosis.

Wang KC et al. reported a major risk factor for EAOC is increasing age [13]. The NHIRD database in Taiwan revealed that the incidence rate of EAOC elevated concurrently with age, ranging from 4.99 per 10,000 person-years in women < 30 years to 35.81 in women < 50 years, thus leading to a progressive increased risk of epithelial ovarian cancer (crude HRs ranging from 2.80 to 6.74 and adjusted HRs ranging from 3.34 to 9.63) compared to age-matched women without endometriosis. The risks of epithelial ovarian cancer of women at > 50 years with endometriosis were significantly higher than those in age-matched women without endometriosis (adjusted HR 9.63) and young women at < 30 years with endometriosis (adjusted HR 4.97).

In this study, there was no positive correlation between increasing age and the miR-486-5p expression in serum and ascites in EAOC. However, the average age of EAOC patients was 52.7 ± 12.4 years, which was significantly higher than that of endometriosis patients. MiR-486-5p has been reported to be involved in different types of cancer including myeloid leukemia [14], gastric adenocarcinoma [15], lung cancer [16–19], colorectal carcinoma [20], pancreatic ductal adenocarcinoma [21], esophageal squamous cell carcinoma [22], hepatocellular carcinoma [23], breast cancer [24], and prostate cancer [25]. However, its function in cancer is still not fully understood. A study on the mechanism of miR-486-5p revealed that the down-regulation of miR-486-5p could inhibit cell migration and invasion in vitro, and metastasis in vivo*,* in lung cancer by targeting genes such as ARHGAP5 [17]. Another study showed that miR-486-5p could inhibit proliferation, migration and invasion of cancer in vitro and suppress hepatocellular carcinoma (HCC) growth in vivo by targeting phosphoinositide-3-kinase regulatory subunit 1 (PIK3R1), which plays an important role in the metabolic actions of insulin. A mutation in this gene has also been associated with insulin resistance [26]. Moreover, the cell growth of papillary thyroid carcinoma is inhibited by miR-486-5p by targeting fibrillin-1 [27], and estrogen receptor-mediated miR-486-5p could regulate the expression of OLFM4 in ovarian cancer [28]. Chunmei Li et al. reported that in cervical cancer miR-486-5p biologically functions through PTEN, which was confirmed as a candidate target of miR-486-5p by the Target Scan database [29]. A dual luciferase reporter assay confirmed that miR-486-5p directly targeted the PTEN 3′-UTR. Previous studies have also shown that the activation of miR-486-5p blocked PTEN, thus leading to Akt phosphorylation in both mice and humans [30]. However, it remains unclear as to how miR-458-5p regulates the development of EAOC. Because there have been no previous studies on miR-486-5p function in EAOC, our objective here was to investigate the biological functions of this miRNA and its potential underlying mechanisms in this disease.

In our study, the ability to proliferate and invade the immortalized endometriotic epithelial cell line was enhanced by the overexpression of miR-486-5p and inhibited by the miR-486-5p receptor inhibitor. Generally, endometriosis-associated ovarian cancer itself is less frequently identified compared with epitherial ovarian cancer.

## Conclusion

The limitations of this study may be due to the small number of cases and that the prognostic outcome could not be determined because they are still being followed up. Moreover, due to the small number of each subtype of ovarian cancer, the difference of miR-486-5p expression in each subtype could not be evaluated. In order to further prospect the malignant potential of endometrial cyst, more studies are necessary to investigate the functional target of miR-486-5p as well as the relationship of miR-486-5p and the prognostic outcome of EAOC.

The miR-486-5p could be an oncogenic miRNA in endometriosis-associated ovarian cancer and a noninvasive biomarker to prospect the severity of ovarian endometrioma.

## Data Availability

The dataset supporting the conclusions of this article is included within the. article.

## Abbreviations

miRNAs: MicroRNAs
EAOC: Endometriosis-associated ovarian cancers
EOC: Epithelial ovarian cancer
OE: Ovarian endometrioma
FBS: Fetal bovine serum
PBS: Phosphate-buffered saline solution

## References

[1] Sampson JA (1925). Endometrial carcinoma of the ovary arising in endometrial tissue in that organ. Arch Surg.

[2] Pearce CL, Templeman C, Rossing MA, Lee A, Near AM, Webb PM (2012). Association between endometriosis and risk of histological subtypes of ovarian cancer: a pooled analysis of case-control studies. Lancet Oncol.

[3] Somigliana E, Vigano’ P, Parazzini F, Stoppelli S, Giambattista E, Vercellini P (2006). Association between endometriosis and cancer: a comprehensive review and a critical analysis of clinical and epidemiological evidence. Gynecol Oncol.

[4] Ren T, Sun TT, Wang S, Sun J, Xiang Y, Shen K, Lang JH (2018). Clinical analysis of chemo-resistance risk factors in endometriosis associated ovarian cancer. J Ovarian Res.

[5] Kurman RJ, Shih IM (2010). The origin and pathogenesis of epithelial ovarian cancer: a proposed unifying theory. Am J Surg Pathol.

[6] Munksgaard PS, Blaakaer J. The association between endometriosis and ovarian cancer: a review of histological, genetic and molecular alterations. Gynecol Oncol 2012;124:164–9. 10.1016/j.ygyno.2011.10.001 22032835

[7] Nothnick WB (2017). MicroRNAs and endometriosis: distinguishing drivers from passengers in disease pathogenesis. Semin Reprod Med.

[8] Cho S, Mutlu L, Grechukhina O, Taylor HS (2015). Circulating microRNAs as potential biomarkers for endometriosis. Fertil Steril.

[9] Rekker K, Tasa T, Saare M, Samuel K, Kadastik Ü, Karro H (2018). Differentially-Expressed miRNAs in Ectopic Stromal Cells Contribute to Endometriosis Development: The Plausible Role of miR-139-5p and miR-375. Int J Mol Sci.

[10] Cosar E, Mamillapalli R, Ersoy GS, Cho S, Seifer B, Taylor HS (2016). Serum microRNAs as diagnostic markers of endometriosis: a comprehensive array-based analysis. Fertil Steril.

[11] Wu RL, Ali S, Bandyopadhyay S, Alosh B, Hayek K, Daaboul MF (2015). Comparative analysis of differentially expressed miRNAs and their downstream mRNAs in ovarian Cancer and its associated endometriosis. J Cancer Sci Ther.

[12] Bono Y, Kyo S, Takakura M, Maida Y, Mizumoto Y, Nakamura M (2012). Creation of immortalised epithelial cells from ovarian endometrioma. Br J Cancer.

[13] Wang KC, Chang WH, Lee WL, Huang N, Huang HY, Yen MS (2014). An increase risk of epitherial ovarian cancer in Taiwanese women with a new surgico-pathological diagnosis of endometriosis. BMC Cancer.

[14] Shaham L, Vendramini E, Ge Y, Goren Y, Birger Y, Tijssen MR (2015). MicroRNA-486-5p is an erythroid oncomiR of the myeloid leukemias of Down syndrome. Blood.

[15] Chen H, Ren C, Han C, Wang D, Chen Y, Fu D (2015). Expression and prognostic value of miR-486-5p in patients with gastric adenocarcinoma. PLoS One.

[16] Hu Z, Chen X, Zhao Y, Tian T, Jin G, Shu Y (2010). Serum microRNA signatures identified in a genome-wide serum microRNA expression profiling predict survival of non-small-cell lung cancer. J Clin Oncol.

[17] Wang J, Tian X, Han R, Zhang X, Wang X, Shen H (2014). Downregulation of miR-486-5p contributes to tumor progression and metastasis by targeting protumorigenic ARHGAP5 in lung cancer. Oncogene.

[18] Peng Y, Dai Y, Hitchcock C, Yang X, Kassis ES, Liu L (2013). Insulin growth factor signaling is regulated by microRNA-486, an underexpressed microRNA in lung cancer. Proc Natl Acad Sci U S A.

[19] Li W, Wang Y, Zhang Q, Tang L, Liu X, Dai Y (2015). MicroRNA-486 as a biomarker for early diagnosis and recurrence of non-small cell lung Cancer. PLoS One.

[20] Liu C, Li M, Hu Y, Shi N, Yu H, Liu H (2016). miR-486-5p attenuates tumor growth and lymphangiogenesis by targeting neuropilin-2 in colorectal carcinoma. Onco Targets Ther.

[21] Mees ST, Mardin WA, Sielker S, Willscher E, Senninger N, Schleicher C (2009). Involvement of CD40 targeting miR-224 and miR-486 on the progression of pancreatic ductal adenocarcinomas. Ann Surg Oncol.

[22] Yi Y, Lu X, Chen J, Jiao C, Zhong J, Song Z (2016). Downregulated miR-486-5p acts as a tumor suppressor in esophageal squamous cell carcinoma. Exp Ther Med.

[23] Youness RA, El-Tayebi HM, Assal RA, Hosny K, Esmat G, Abdelaziz AI (2016). MicroRNA-486-5p enhances hepatocellular carcinoma tumor suppression through repression of IGF-1R and its downstream mTOR, STAT3 and c-Myc. Oncol Lett.

[24] Zhang G, Liu Z, Cui G, Wang X, Yang Z (2014). MicroRNA-486-5p targeting PIM-1 suppresses cell proliferation in breast cancer cells. Tumour Biol.

[25] Zhang X, Zhang T, Yang K, Zhang M, Wang K (2016). miR-486-5p suppresses prostate cancer metastasis by targeting snail and regulating epithelial-mesenchymal transition. Onco Targets Ther.

[26] Huang XP, Hou J, Shen XY, Huang CY, Zhang XH, Xie YA (2015). MicroRNA-486-5p, which is downregulated in hepatocellular carcinoma, suppresses tumor growth by targeting PIK3R1. FEBS J.

[27] Ma X, Wei J, Zhang L, Deng D, Liu L, Mei X (2016). miR-486-5p inhibits cell growth of papillary thyroid carcinoma by targeting fibrillin-1. Biomed Pharmacother.

[28] Ma H, Tian T, Liang S, Liu X, Shen H, Xia M (2016). Estrogen receptor-mediated miR-486-5p regulation of OLFM4 expression in ovarian cancer. Oncotarget.

[29] Li C, Zheng X, Li W, Bai F, Lyu J, Meng QH (2018). Serum miR-486-5p as a diagnostic marker in cervical cancer: with investigation of potential mechanisms. BMC Cancer.

[30] Matthew S, Alexander JCC, Motohashi N, Vieira NM, Eisenberg I (2014). MicroRNA-486–dependent modulation of DOCK3/PTEN/AKT signaling pathways improves muscular dystrophy–associated symptoms. J Clin Invest.

